# Mitigation of oxidative stress and inflammatory factors, along with the antibrowning and antimicrobial effects of cassia seed microbial fermentation solution

**DOI:** 10.3389/fmicb.2024.1400505

**Published:** 2024-05-09

**Authors:** Haohui Xie, Quliang Gu, Weiji Chen, Xiangyu Meng, Zhenyu Guo, Yue Zhang, He Li

**Affiliations:** ^1^School of Basic Medical Sciences, Guangdong Pharmaceutical University, Guangzhou, China; ^2^Qingdao Benyue Biotechnology Co., Ltd, Qingdao, China

**Keywords:** zebrafish, cassia seed, inflammatory model, chorioallantoic membrane, yeast, fluorescent probe

## Abstract

**Introduction:**

Cassia seeds, originating from the mature seeds of leguminous cassia species, possess pharmacological effects attributed to their rich composition of various active ingredients, notably anthraquinones. While current research predominantly focuses on pharmaceutical extractions, there has been limited progress in fermentation studies.

**Methods:**

Our study aimed to enhance the content of active compounds such as anthraquinones, flavonoids, and polyphenols using microbial fermentation techniques. We specifically optimized a fermentation system through a single-factor experimental design.

**Results:**

The antioxidant properties of the fermentation solution were validated through assays involving HaCaT cells and zebrafish. We observed effective suppression of inflammatory reactions in both RAW264.7 cells and transgenic zebrafish by the fermentation solution. Moreover, significant inhibition of tyrosinase activity and melanin production was evident in B16-F10 cells and zebrafish. Positive outcomes were also obtained in antibacterial assays and chick embryo experiments.

**Discussion:**

These findings highlight the potential of cassia seed fermentation solution as a safe and eco-friendly material in food chemistry and biomedical sciences.

## 1 Introduction

Cassia seed represents the dried mature seed of the leguminous cassia or cassia minor. Recent pharmacological studies have unveiled a spectrum of biological activities associated with cassia seeds and their constituents. These activities encompass promoting bowel movement, liver protection, neuroprotection, immune modulation, anti-inflammatory (Zhao et al., 2024), and antioxidant effects (Khodeer et al., 2022). Furthermore, they exhibit antibrowning and antimicrobial properties (Aqawi et al., 2021), along with activities in reducing blood lipids and managing diabetes-related complications (Jia et al., 2023). A myriad of components, such as anthraquinones, naphthalenes, flavonoids, polysaccharides, and others, have been detected in cassia seeds (Chen et al., 2023). Yeast, a single-celled eukaryotic organism prevalent in the environment, serves as a probiotic in the traditional fermentation of foods and beverages (Riesute et al., 2021). However, yeast’s potential as a probiotic in traditional fermentation remains understudied, with only a few researchers reviewing its potential in food fermentation (Sambrani et al., 2021).

Fermentation involves various advantageous biological and natural chemical alterations in plants due to microbial processes, aiding in transforming active compounds and boosting microbial transformation of these elements (Zhao et al., 2021; Zhou et al., 2022). Utilizing microbial fermentation techniques can transform large molecules in raw materials into smaller, more easily absorbed molecules, thus improving the utilization of active ingredients (Li et al., 2021). Additionally, microorganisms have the capability to transform certain natural substances into more active or stable forms, thereby heightening their pharmacological efficacy. For instance, Lee et al. demonstrated the augmentation of antioxidant properties in oat-derived active compounds through bacterial conversion (Lee et al., 2022). Meanwhile, Yan et al. delved further into the potential synergistic effects of liquid fermentation technology, combining fermented products with other food components to enhance their nutritional value and biological activity (Yan et al., 2023). Furthermore, some researchers have delved into the mechanisms through which microbial fermentation impacts the levels of active constituents in traditional Chinese medicine (Zhou et al., 2023; Xu et al., 2024), suggesting that fermentation serves as an effective means to bolster their functional components. The aforementioned research demonstrates that microbial fermentation technology opens up new avenues and possibilities for drug development and utilization, offering vast prospects and significant importance in pharmaceutical research and applications.

Methods to increase the concentration of metabolites in pharmaceutical fermentation solution include optimizing fermentation conditions, such as adjusting temperature and pH, and adding appropriate nutrients or auxiliary substances to promote microbial growth and the production of metabolites. Additionally, using different types or strains of microorganisms for fermentation may lead to the generation of different metabolites and potentially higher levels of active ingredients. Apart from microbial fermentation, other approaches such as optimizing extraction techniques (Lama-Munoz and Contreras, 2022), chemical synthesis, genetic engineering (Bijukumar and Somvanshi, 2024), or selective breeding can also be employed to achieve the desired outcome. This study involves the microbial fermentation of cassia seeds using yeast, augmenting the levels of active components in the fermentation solution. The study aims to enhance its application in various fields of food chemistry and medicine, serving as an antioxidant, anti-inflammatory agent, antibacterial agent, and tyrosinase inhibitor, providing innovative scientific and engineering solutions for a sustainable future for both humans and nature.

## 2 Materials and methods

### 2.1 Test materials

The yeast strains (YF1401, YF1036, YF1201, Y101, YF1001, Y73, YF1301, YF1520, Y100, YF1301, YF3045, YF1270), *Staphylococcus aureus*, methicillin-resistant *Staphylococcus aureus* (MRSA), *Escherichia coli*, and *Staphylococcus epidermidis*, 2,3,5-triphenyltetrazolium chloride (TTC) with a purity of ≥98%, Human immortalized keratinocytes (HaCaT), murine melanoma cells (B16-F10), RAW264.7 macrophages, SPF-grade fertilized chicken eggs, were all purchased from Guangdong HuanKai Microbial Science and Technology Co., Ltd. AB strain zebrafish and transgenic zebrafish Tg (lyzc:dsred) were provided by the China Zebrafish Resource Center. Superoxide Dismutase (SOD) assay kit (Product No: A001-3, WST-1 method), Malondialdehyde (MDA) assay kit (Product No: A003-1, TBA method), Total Antioxidant Capacity (T-AOC) assay kit (Product No: A015-2-1, ABTS method), Catalase (CAT) assay kit (Product No: A007-1-1, ammonium molybdate method), MTT cell proliferation and cytotoxicity assay kit, were all purchased from the Institute of Biomedical Engineering, Nanjing, China. The Bradford protein concentration determination kit (Product No: P0006) and reactive oxygen species assay kit (Product No: S0033S) were purchased from Beyotime Biotechnology Company, China.

### 2.2 Screening of dominant fermentation strains

#### 2.2.1 Preparation of yeast seed liquor

The yeast strain was inoculated into LB medium under sterile conditions and incubated at 37°C with agitation at 220 rpm for one day. The bacterial concentration was measured using a spectrophotometer to achieve an OD_600 nm_ reading between 0.8 and 1.2 (Khoury et al., 2020). The yeast strains were cryopreserved using a 30% glycerol-sodium chloride solution and stored at −80°C.

#### 2.2.2 Preparation of cassia seed fermentation solution

The dried mature cassia seeds are ground in a pulverizer and then subjected to sterilization at 121°C under high pressure for 20 min prior to use. The fermentation composition comprises cassia seeds, yeast, and sterile distilled water. Fermentation is conducted under conditions of 37°C and 220 rpm for 48 h, evaluating anthraquinones, polysaccharides, flavonoids, polyphenols, and ABTS clearance rate as assessment indices to select the yeast strain most suitable for cassia seed fermentation.

#### 2.2.3 Standard curve preparation and active ingredient determination

Liu et al. employed UV spectroscopy analysis to determine the total anthraquinone content in water extracts of cassia seeds treated by various methods (Liu et al., 2023). The method was optimized according to experimental requirements for the quantification of anthraquinone content in fermented cassia seed solutions. The operational steps of this method are outlined as follows: The standard stock solution of 1,8-dihydroxyanthraquinone was completely dissolved in methanol, and a calibration curve was constructed. Specimens were made up to 5 mL and reacted with a 0.5% magnesium acetate-methanol solution for 5 min. After centrifugation, the supernatant was taken, and its absorbance was measured using a 514-nanometer wavelength.

In their study on “*Morinda citrifolia* L. (Noni) polysaccharides at different stages of maturity,” Cai et al. utilized the phenol-sulfuric acid method with glucose as the standard for determining polysaccharide content (Cai et al., 2023). Subtle modifications were made to the experimental method based on this approach. Each concentration of the standard or sample (0.5 mL), followed by the addition of 0.5 mL of 5% phenol and 2.5 mL of concentrated sulfuric acid. After 10 min of settling at room temperature, the mixture was placed in a constant-temperature water bath at 30°C for 30 min. Upon centrifugation, the supernatant was collected, and its absorbance was measured at 490 nanometers.

In the fermentation of Lactobacillus by Wei et al., flavonoid content was determined using rutin as a standard substance, employing the colorimetric analysis via UV spectrophotometry (Wei et al., 2023). With minor adjustments, 0.5 mL of 5% NaNO2 solution and 5% Al(NO3)3 solution were added to the sample and allowed to react for 6 min. Subsequently, 3 mL of 4% NaOH solution was added, followed by thorough mixing and a 10-min incubation period. After centrifugation, the supernatant was collected for measurement. The assessment of polyphenol levels was conducted with Gallic acid serving as the reference compound. Employing the Folin–Ciocalteu method (Richard-Dazeur et al., 2023) with slight modifications. The ABTS method was utilized to evaluate the scavenging capability of cassia seed fermentation solution against free radicals (Aziz et al., 2023). ABTS clearance rate calculation see Equation (1).


(1) $$\mathrm{ABTS}\;\mathrm{clearance}\;\mathrm{rate}\;\left(\%\right)=\frac{A_{0}-A_{1}}{A_{0}}\times 100\%$$

Here, A_0_ represents the absorbance from the interaction of the ABTS liquid with ethanol, while A_1_ signifies the absorbance from the interaction of the ABTS liquid with the specimen.

### 2.3 Improved of fermentation parameters

#### 2.3.1 Single factor design

Under the fermentation settings maintained at 37°C and 220 rpm for 48 h, with anthraquinone content as the evaluative criterion, single-factor experiments were conducted to ascertain the influence of carbon source, carbon source concentration, inoculum volume, material-liquid ratio, fermentation temperature, and fermentation duration on anthraquinone content. Each experiment was conducted three times.

#### 2.3.2 Plackett-Burman design

A single-factor experimental setup using the Plackett-Burman design was executed through Design-Expert 13 software. This aimed to identify key elements influencing the anthraquinone levels in cassia seed fermentation solution. By evaluating both high (+) and low (−) levels of these factors against the cassia seed anthraquinone content, each group underwent three parallel experiments.

#### 2.3.3 Box-Behnken experimental design

In light of the findings from the Plackett-Burman experiment, three primary influencing factors affecting anthraquinone content were identified. Using anthraquinone content as the response variable, an optimization experiment with *N* = 17 was designed to investigate the interactions among key factors, aiming to simulate and deduce the optimal fermentation conditions. Each group underwent three parallel experiments.

### 2.4 *In vitro* antioxidant cell experiments

#### 2.4.1 Safe concentration of fermentation solution with H_2_O_2_

The human immortalized keratinocytes (HaCaT) were cultured in a high-glucose DMEM medium supplemented with a 1% penicillin–streptomycin mixture and 10% fetal bovine serum (FBS). Cells were seeded at a concentration of 1 × 10^5^/ml in a 96-well plate and left to incubate for 24 h.

Various concentrations of cassia seed fermentation solution and H_2_O_2_ were applied to the cells. The MTT assay, which relies on the reduction of MTT by dehydrogenases within cell mitochondria to form a purple formazan precipitate, was used to evaluate the safe concentration range of the fermentation solution and H_2_O_2_ in cells (Jalil Sarghaleh et al., 2023). Cell viability rate is calculated using Equation (2).


(2) $$\mathrm{Cell}\;\mathrm{viability}\;\left(\%\right)=\frac{\left(A_{1}-A_{0}\right)}{\left(A_{2}-A_{0}\right)}\times 100\%$$

Where A_1_ represents the absorbance reading of the test group, A_0_ stands for the absorbance reading of the blank group, and A_2_ denotes the absorbance reading of the control group.

#### 2.4.2 Modeling of oxidative damage in HaCaT cells

Cells were seeded into a 6-well cell culture plate at a concentration of 1 × 10^5^/ml and left to grow for 72 h. The experiment involved a control group (DMEM basal medium), a model group (DMEM basal medium), a positive control group [25 μg/mL vitamin C (VC)], and fermentation solution group (with low, medium and high concentrations of cassia seed fermentation solution). After an additional 24 h of incubation, 0.156 mmol/L H_2_O_2_ solution was added to all groups except the control group. After a 4 h treatment, cells were harvested using trypsin and collected in PBS. The cells were harvested through trypsin digestion using PBS as the medium. Subsequently, the collected cells were sonicated at 4°C (5 min, 100 W power, 5 s/frequency, 20 s interval) to generate cellular lysates.

#### 2.4.3 Measurement of HaCaT cell-related content

The specific steps for protein standard curve and sample content determination are as follows: 5 μL of various concentrations of the protein standard or samples are added to 250 μL per well of G250 staining solution, followed by measurement at OD_570nm_. According to the assay kit instructions, the determination of superoxide dismutase (SOD), malondialdehyde (MDA), total antioxidant capacity (T-AOC), and catalase (CAT) content is performed within the cell lysate. The reference formulas for relevant calculations are as follows Equations (3–6).


(3) $$\begin{array}{l} \;\mathrm{SOD}\;\mathrm{inhibition}\;\mathrm{rate}\;\left(\%\right)=\\ \;1-\frac{\left(\mathrm{Aassay}-\mathrm{Acontrol}\;\mathrm{blank}\right)}{\left(\mathrm{Acontrol}-A\;\mathrm{assay}\;\mathrm{blank}\right)}\times 100\% \end{array}$$

(4) $$\begin{array}{l} \;\mathrm{SOD}\;\mathrm{activity}=\frac{\mathrm{SOD}\;\mathrm{inhibition}}{50\%}\\ \;\times \frac{\mathrm{reaction}\;\mathrm{system}\;\left(0.24\mathrm{mL}\right)}{\mathrm{dilution}\;\mathrm{factor}\;\left(0.02\mathrm{mL}\right)}÷\mathrm{protein}\;\mathrm{content} \end{array}$$

(5) $$\begin{array}{l} MDA\;\mathrm{content}=\\ \frac{\left(A\;\mathrm{assay}-A\;\mathrm{Control}\right)\times \mathrm{Standard}\;\mathrm{Concentration}}{A\;\mathrm{Standard}-A\;\mathrm{Blank}}÷\mathrm{protien}\;\mathrm{content} \end{array}$$

(6) CATviability=ΔA×273÷Vsample÷60÷proteincontent

#### 2.4.4 Determination of intracellular reactive oxygen species (ROS) content

According to the procedures outlined in section 2.4.2, cells were grouped and treated accordingly. A 0.156 mmol/L H_2_O_2_ solution was added to all groups except the blank group. After a 4-h reaction period, a final concentration of 10 μmol/L fluorescent probe DCFH-DA was added. Subsequently, the cells were incubated at 37°C in the dark for 40 min. Finally, the fluorescence intensity of cells in each group was observed using a fluorescence microscope, and images were captured for documentation.

### 2.5 *In vivo* antioxidant zebrafish experiments

#### 2.5.1 Effect of cassia seed fermentation solution on zebrafish embryos

The zebrafish experiment utilized wild-type AB strain zebrafish to simulate optimal zebrafish growth conditions, maintaining a water temperature of 28°C and a light–dark cycle of 14:10. Zebrafish reproduction followed a mature ratio of females to males at 1:2 to acquire zebrafish embryos. Only normally fertilized embryos were selected for experimentation. The zebrafish embryos were exposed to varying concentrations of cassia seed fermentation solution and 2, 2′-Azobis (2-methylpropionamidine) dihydrochloride (AAPH). The developmental progression of these embryos was observed under a stereomicroscope for 72 h, and formulas were utilized to compute the embryo survival rate, hatching rate, and malformation rate. This methodology aimed to screen for the safe concentration of the fermentation solution and the stimulating concentration of AAPH. See Equations (7–10) for relevant calculations.


(7) $$\mathrm{Survival}\;\mathrm{rate}\;\left(\%\right)=\frac{\mathrm{number}\;\mathrm{of}\;\mathrm{surviving}\;\mathrm{embryos}}{\mathrm{total}\;\mathrm{number}\;\mathrm{of}\;\mathrm{embryos}}\times 100\%$$

(8) $$\mathrm{Hatching}\;\mathrm{rate}\;\left(\%\right)=\frac{\mathrm{number}\;\mathrm{of}\;\mathrm{hatched}\;\mathrm{embryos}}{\mathrm{total}\;\mathrm{number}\;\mathrm{of}\;\mathrm{embryos}}\times 100\%$$

(9) $$\begin{array}{l} \mathrm{Malformation}\;\mathrm{rate}\;\left(\%\right)=\\ \;\frac{\left(\mathrm{Number}\;\mathrm{of}\;\mathrm{malfomed}\;\mathrm{and}\;\mathrm{dead}\;\mathrm{embryos}\right)}{\mathrm{total}\;\mathrm{number}\;\mathrm{of}\;\mathrm{embryos}}\times 100 \end{array}$$

#### 2.5.2 Modeling of zebrafish oxidation

The experimental setup consisted of a control group (zebrafish culture medium), a model group (zebrafish culture medium), a positive control group (10 μg/mL vitamin C), and fermentation solution groups (low, medium and high concentrations of cassia seed fermentation solution), each containing 20 zebrafish embryos. After culturing at 28°C for 72 h, a 0.6 mmol/L AAPH solution was added to each group, excluding the control group. Following a 6 h exposure period, zebrafish from each group were sonicated in distilled water as a medium. The resultant homogenates were centrifuged to collect the supernatant for assessing SOD, MDA, T-AOC, and CAT levels within the zebrafish.

#### 2.5.3 Determination of ROS content in zebrafish

Selecting normally developed, non-malformed AB strain zebrafish at 3 days post-fertilization, placing 10 fish per group in a 24-well plate, with each experimental setup conducted in triplicate. The experimental design comprises a control group (zebrafish culture medium), a model group (zebrafish culture medium), a positive control group (10 μg/mL vitamin C), and fermentation solution groups (low, medium and high concentrations of cassia seed fermentation solution). After a 2 h incubation, 10 mmol/L copper sulfate solution was added to each group except the control group. Following a 30 min reaction, a final concentration of 10 μmol/L fluorescent probe DCFH-DA was introduced, incubating at 28°C in the dark for 40 min. Subsequently, the fluorescent intensity of the zebrafish in each group was observed using a fluorescence microscope.

### 2.6 Anti-inflammatory cell experiments

#### 2.6.1 Safe concentration of fermentation solution for RAW264.7 cells

The logarithmically growing RAW264.7 macrophages were introduced into a 96-well cell culture plate at a concentration of 1 × 10^5^ cells/ml and incubated for 24 h (Xu et al., 2023). DMEM served as the dilution medium, the fermented solution of cassia seeds was diluted to various concentrations and applied to the cells for 24 h. The MTT assay determined cell viability to establish the safe concentration range. Each concentration group was subjected to six parallel experiments to ensure reliability and reproducibility of results.

#### 2.6.2 Establishment of the RAW264.7 cell anti-inflammatory model

The logarithmically growing RAW264.7 cells were introduced into a 96-well plate at a concentration of 1 × 10^5^ cells/mL and cultured for 24 h. The experimental setup included a control group (DMEM basal culture medium), a model group (1 mg/mL lipopolysaccharide (LPS)), a positive group (100 μg/mL dexamethasone and 1 mg/L LPS), and fermentation solution group (low, medium and high doses of cassia seed fermentation solution and 1 mg/L LPS). Following a 24 h incubation, cell morphology changes were captured by microscopy. Subsequently, the cells were lysed by repeated freeze-thawing under cryogenic conditions, centrifuged and the supernatant was taken to detect the TNF-α and NO content in them.

### 2.7 Fermentation solution anti-inflammatory zebrafish experiments

#### 2.7.1 Effect of fermentation solution on transgenic zebrafish

Transgenic zebrafish Tg (lyzc:dsred) at 3 dpf (days post-fertilization) were selected for the experiment. This strain is characterized by red fluorescence in neutrophils and macrophages. The experiments were arranged with 10 individuals in each group, and three parallel treatments were conducted for each experimental group, which were placed in 24-well plates. The survival rate of these zebrafish was observed after 24 h of incubation in different concentrations of cassia seed fermentation solution using stereomicroscopy.

#### 2.7.2 Copper sulfate-induced inflammation model

The experimental design included a control group (zebrafish culture medium), a model group (zebrafish culture medium), a positive group (dexamethasone 10 μg/mL), and fermentation solution group (low, medium and high concentrations of cassia seed fermentation solution). After a 2 h exposure, each group, except the control group, was treated with copper sulfate at a concentration of 10 mmol/L. Following a 30-min reaction period, the migration of inflammatory cells was captured under a fluorescence microscope, and images were recorded for documentation. The Image J application was utilized to quantify the quantity of inflammatory cells within the identical area of the neural thalamus.

#### 2.7.3 Tail cutting-induced inflammation model

After anesthetizing zebrafish larvae in all groups except the control with 0.02% tricaine, tail fins were surgically removed under a microscope. The experimental groups included a control group (zebrafish culture medium), a model group (zebrafish culture medium), a positive control group (dexamethasone 10 μg/mL), and fermentation solution group (low, medium and high concentrations of cassia seed fermentation solution). Following a 6 h exposure, the aggregation of macrophages and neutrophils at the site of zebrafish tail injury was observed under a fluorescence microscope. The Image J software was employed to quantify the number of inflammatory cells in the same region.

### 2.8 Antibrowning cell experiments

#### 2.8.1 Safe concentrations of fermentation solution for B16-F10 cells

Mouse melanoma cells (B16-F10) were cultured in RPMI-1640 medium supplemented with 1% penicillin–streptomycin mixture and 10% fetal bovine serum (FBS) (Gajić et al., 2022). The cells were placed in a 96-well plate at a density of 1 × 10^5^ cells/ml and left to incubate for 24 h. After discarding the supernatant, different concentrations of Cassia seed fermentation solution were added. The cells were subsequently incubated for 24 h, and their viability was assessed via the MTT test to determine the acceptable concentration range of the fermentation solution.

#### 2.8.2 Measurement of tyrosinase and melanin content in cells

The B16-F10 cells were introduced at a density of 1 × 10^5^ cells/ml in a six-well cell culture plate with RPMI-1640 medium and cultured for 72 h. The supernatant was discarded, and the culture medium was substituted with DMEM medium to promote cell differentiation and melanin synthesis. The experimental groups consisted of a control group (DMEM basal culture medium), a positive group (250 μg/mL kojic acid), and fermentation solution group (with low, medium, and high concentrations of cassia seed fermentation solution). Following an additional 24 h of incubation, the cellular melanin conditions were observed and documented using a microscope. The cells were enzymatically dissociated using trypsin, followed by ultrasonic disruption. Supernatant collection after centrifugation enabled the determination of intracellular tyrosinase activity via the L-DOPA method (Goenka et al., 2021). Simultaneously, the intracellular melanin content was quantified using the NaOH lysis method (Bodurlar and Caliskan, 2022; Hong et al., 2023). See Equations (10, 11) for relevant calculations.


(10) $$\mathrm{Tyrosinase}\;\mathrm{inhibition}\;\mathrm{rate}\;\left(\%\right)=1-\frac{A_{1}-A_{0}}{A_{2}-A_{0}}\times 100\%$$

(11) $$\mathrm{Melanin}\;\mathrm{inhibition}\;\mathrm{rate}\;\left(\%\right)=1-\frac{A_{1}-A_{0}}{A_{2}-A_{0}}\times 100\%$$

Where A_1_ represents the OD value of the test group, A_2_ is the OD value of the blank group, and A_0_ stands for the OD value of the culture medium.

### 2.9 Antibrowning zebrafish experiments

Fertilized embryos of zebrafish of the AB strain were placed in 6-well plates, 20/well. The experimental conditions were maintained at 28°C under a 14:10 light–dark cycle. Experimental groups included the control group, a positive group (250 μg/mL kojic acid), and fermentation solution group (low, medium, high concentrations of cassia seed fermentation solution). After a 72-h incubation period, the distribution of melanin in zebrafish was observed under a microscope, and images were captured for documentation. Subsequently, the zebrafish were sonicated, and their tyrosinase content was determined using the L-DOPA method, while the intracellular melanin content was determined using the NaOH lysis method. Each experimental group was repeated six times in parallel experiments.

### 2.10 Antibacterial efficacy of cassia seed fermentation solution

#### 2.10.1 Determination of minimum inhibitory concentration (MIC)

The bacterial strains were inoculated onto solid culture media using an inoculation loop. After cultivation, a single colony was selected to formulate a seed solution, which was then diluted to a density of 1.5 × 10^8^ CFU/mL. The experimental design included a control group (sterile physiological saline), a negative group (sterile physiological saline + bacterial suspension), a positive group (minocycline + bacterial suspension), and experimental groups (different concentrations of cassia seed fermentation solution + bacterial suspension). After 24 h of incubation, the minimum inhibitory concentration (MIC) was ascertained through colorimetry using the 2,3,5-triphenyltetrazolium chloride (TTC) method (Tanaka et al., 2021). Each experimental condition was repeated three times.

#### 2.10.2 Determination of the ring of inhibition

Under aseptic conditions, spread the bacterial suspension with a density of 1.5 × 10^8^ CFU/mL evenly onto solid culture medium. The experimental design includes a control group, a positive group (minocycline), and a fermentation solution group (low, medium, high concentrations of cassia seed fermentation solution corresponding to each bacterial strain). Affixing drug-sensitive paper discs onto solid medium using tweezers, the culture plates were inverted and kept in a 37°C incubator for 24 h. The diameter of the inhibition zones was subsequently measured using vernier calipers, with each experimental group subjected to three repetitions.

### 2.11 Safety evaluation of fermentation solution

#### 2.11.1 Effects of positive and reference control on chorioallantoic membrane (CAM)

The experiment utilized SPF-grade fertilized chicken embryos from White Leghorn chickens. The embryos were incubated under conditions of 37 ~ 38°C and 60% ~ 70% humidity until the seventh day. Using tweezers, the air chamber portion of the eggshell was removed, and after moistening the egg membrane with physiological saline, it was gently peeled away. Clear and transparent areas of CAM blood vessels were selected and encircled with silicone rubber rings. The positive control involved the use of NaOH solution, while the reference control utilized a mixture of fatty alcohol ether sodium sulfate (Texapon ASV). After a 5-min reaction, the groups were observed for any signs of bleeding, coagulation, or blood vessel lysis.

#### 2.11.2 Effect of fermentation solution on CAM

The degree of stimulation on CAM by cassia seed fermentation extract was evaluated for safety. Each experimental group consisted of 6 chicken embryos, including a control group (physiological saline) and a fermentation solution groups (varied concentrations of cassia seed fermentation solution). Select clear and transparent areas of CAM blood vessels and place silicone rubber rings. After 5 min of action time, bleeding, coagulation and vascular melting were observed in every group. The endpoint score (ES) is classified as follows: ES ≤ 12 indicates no/light irritancy, 12 < ES < 16 indicates moderate irritancy, and ES ≥ 16 indicates strong irritancy/corrosiveness. Evaluate the experimental results based on Supplementary Table S1.

Terminal Score (ES) Calculation Reference Equation (12):


(12) $$ES=\frac{\begin{array}{l} \mathrm{Sum}\;\mathrm{of}\;\mathrm{bleeding},\mathrm{coagulation},\mathrm{and}\;\mathrm{vascular}\;\\ \;\mathrm{lysis}\;\mathrm{degrees}\;\mathrm{in}\;6\;\mathrm{chicken}\;\mathrm{embryos} \end{array}}{3}$$

### 2.12 Data analysis

All experimental data are presented as mean ± standard deviation. Single-factor analysis of variance (ANOVA) and post-hoc multiple comparisons were conducted using GraphPad Prism software. The results were considered significant at *p* < 0.05.

## 3 Results and discussion

### 3.1 Screening of strains

**Figure 1:**
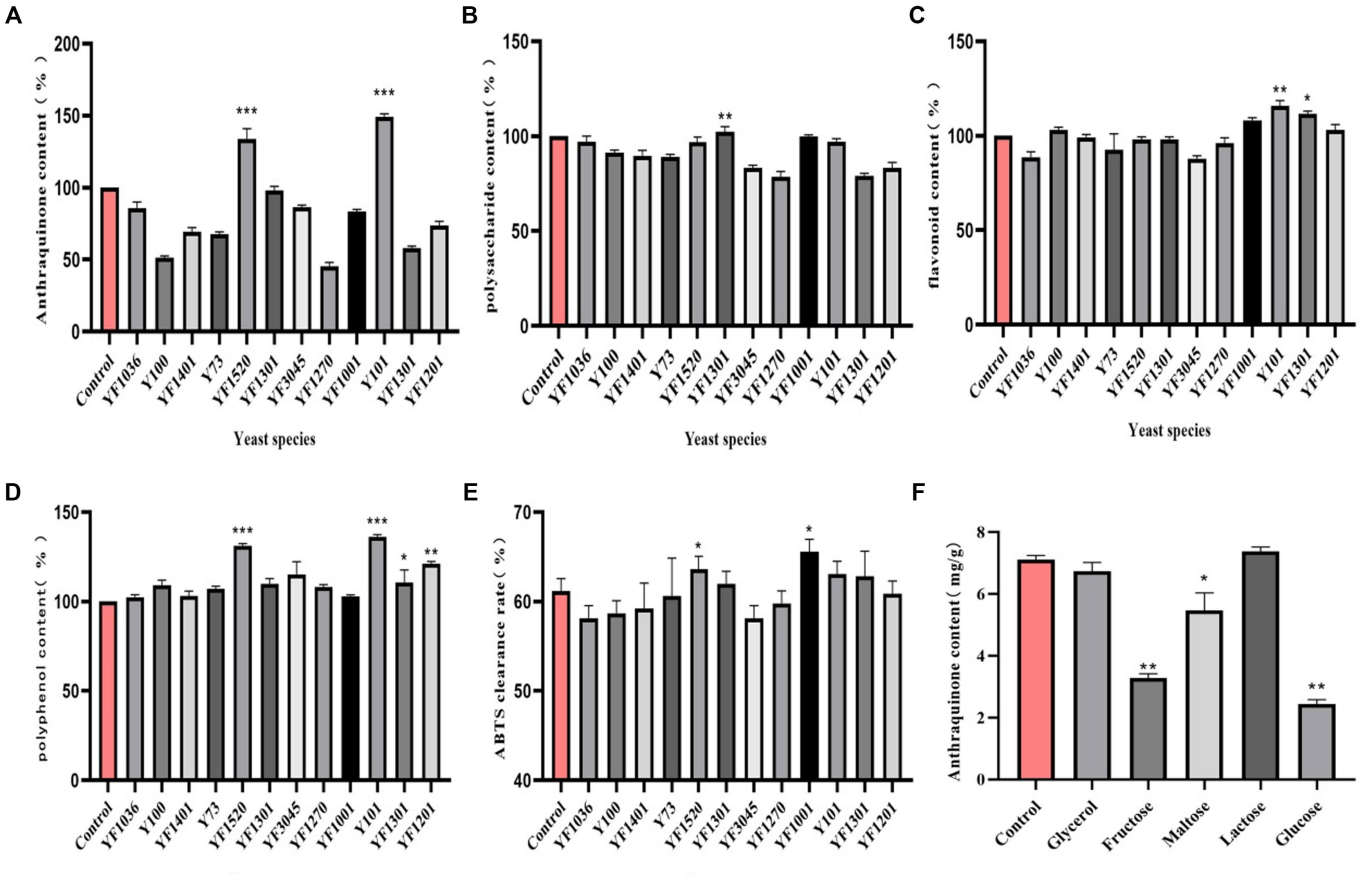
Screening of dominant fermenting strains. **(A)** Anthraquinone content, **(B)** Polysaccharide content, **(C)** Flavonoid content, **(D)** Polyphenol content, **(E)** ABTS clearance rate, **(F)** Carbon source type.

In the fermentation solution of yeast strain Y101, the content of anthraquinone increased by 149% (*p* < 0.01) compared to the control group (100%) (Figure 1A). Due to the conversion of sugars in the fermentation liquid into carbon dioxide and ethanol by yeast (Parapouli et al., 2020), there was no significant increase in polysaccharide content (Figure 1B). According to the Rutin standard curve, it was determined that yeast strain Y101 promoted a 114% (*p* < 0.01) increase in flavonoid content in the fermentation liquid (Figure 1C). The polyphenol content was determined using the Gallic acid standard curve, as yeast metabolites produced during fermentation, such as acetic acid and acetaldehyde, react with phenolic compounds (Szekelyhidi et al., 2022). The results indicate that yeast strain Y101 resulted in a significant 136% increase (*p* < 0.001) in polyphenol content in the fermentation solution compared to the control group (Figure 1D). During fermentation, the hydrolysis of cell wall components and the release of active compounds affected antioxidant activity (Akan, 2022), with yeast strain Y101 displaying an ABTS clearance rate of 63%, higher than the control group (Figure 1E). In conclusion, yeast strain Y101, as the dominant strain in cassia seed fermentation, enhances various effective components. Among them, anthraquinones, flavonoids, and polyphenols exhibit notable antioxidant and anti-inflammatory effects (Liu et al., 2020; Wang et al., 2023).

### 3.2 Improved of fermentation parameters

#### 3.2.1 Results of single factor analysis

**Figure 2:**
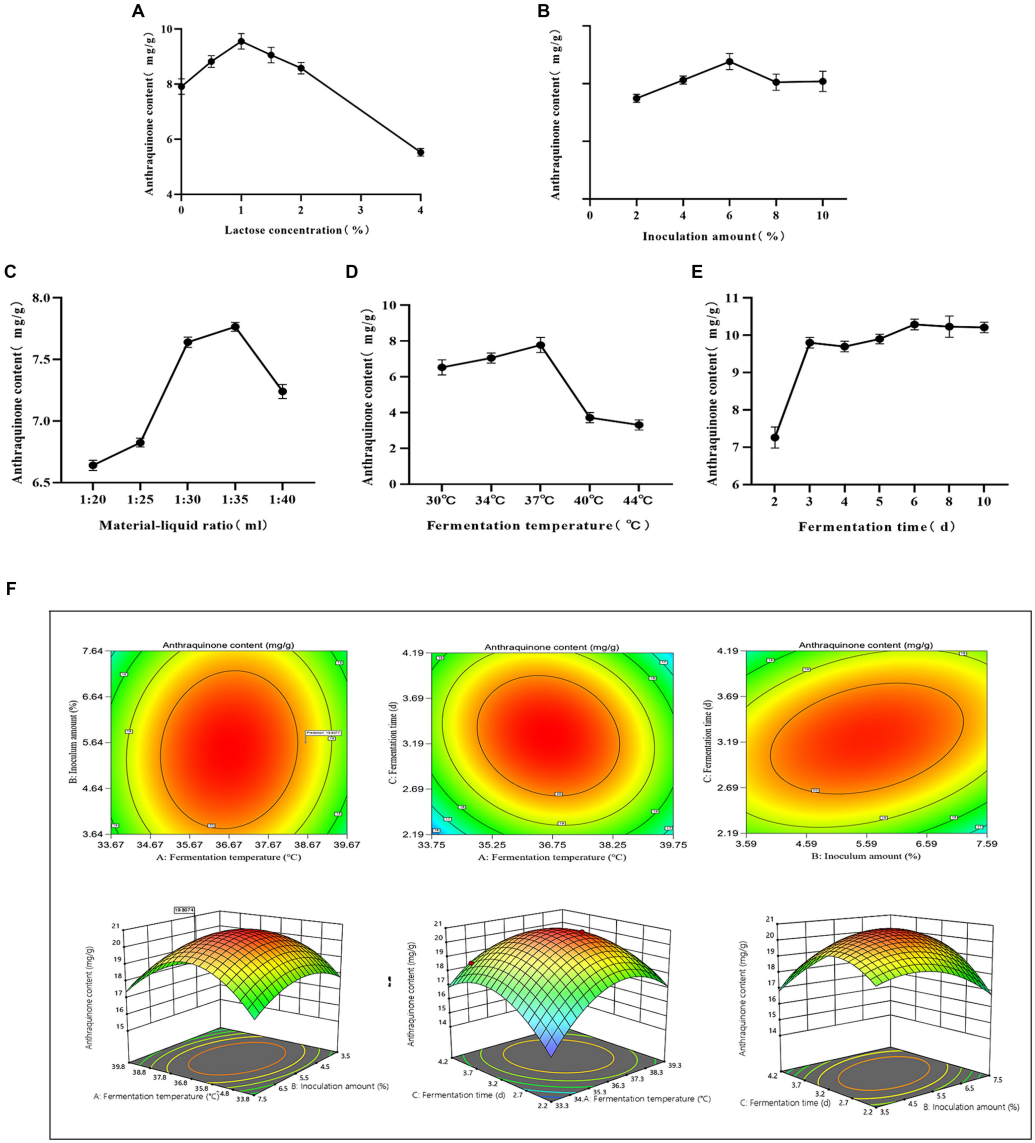
Optimized fermentation. **(A)** Lactose concentration, **(B)** Inoculum amount, **(C)** Material-liquid ratio, **(D)** Fermentation temperature, **(E)** Fermentation time, **(F)** Contour plots and response surfaces.

Compared to other groups, the anthraquinone content increased in the lactose group fermentation solution (Figure 1F). This can be attributed to the carbon source, which directly influences microbial activity and regulates the energy used for bacterial growth (Pan et al., 2023). The maximum anthraquinone content was attained when the lactose concentration was 1% (w/v) (Figure 2A). Inoculation size is crucial for the initial growth capability of microbial populations, and studying various inoculation sizes is essential for achieving high yields (Zhang et al., 2022). The anthraquinone content reached its maximum when the inoculation size (in comparison with the overall volume of the fermentation system) was 6% (Figure 2B). Microbial growth and metabolic processes require a certain amount of moisture, and the maximum increase in anthraquinone content was observed when the material-liquid ratio was 1:35 for fermentation (Figure 2C). Excessive fermentation temperature can inhibit microbial activity, and 37°C is considered optimal (Figure 2D). After 3 days of fermentation, the release of effective components during the fermentation process is essentially complete (Figure 2E), and the anthraquinone content tends to reach a balanced state.

#### 3.2.2 Plackett-Burman experiment results

Using Design-Expert 13 software, a Plackett-Burman experimental design was applied to single-factor conditions, including lactose concentration, inoculation size, fermentation temperature, material-liquid ratio, and fermentation days. The response variable was the anthraquinone content in cassia seed. The resulting multivariable linear equation is expressed as R = +23.65 + 0.1717A – 0.7817B + 1.92C – 0.1050D + 0.5583E, where R represents the anthraquinone content. The coefficient of determination R^2^ for the equation is 0.9522, as determined by regression analysis, indicating the model’s significance within a 95% confidence interval. Comprehensive ranking of factors affecting the response variable is as follows: fermentation temperature > Inoculum amount > fermentation time > lactose concentration > material-liquid ratio. The findings of the experiments are displayed in Supplementary Table S2.

#### 3.2.3 Box–Behnken experiment results

Utilizing multivariate linear regression and polynomial fitting with the available data, the equation model obtained is as follows:

R = +20.57–0.3513A-0.6912B + 1.15C + 0.2725AB-0.6 AC + 0.885 BC-2.41A2-1.1B2-1.91C2. The regression variance analysis reveals a determination coefficient R^2^ = 0.9881, indicating the reasonableness and reliability of the model. The *p*-value for the AB term is 0.1598, suggesting that the interaction effect between fermentation temperature and inoculation size is not significant. However, the *p*-values for the AC and BC terms are both less than 0.05, indicating the presence of interaction effects between fermentation temperature and fermentation days, as well as inoculation size and fermentation days. Detailed results can be found in Supplementary Table S3, and the experimental contour plots and response surfaces are illustrated in Figure 2F. The experimentally simulated optimal fermentation conditions are a temperature of 38.6°C, an inoculation size of 5.64%, and a fermentation duration of 3.2 days. The predicted anthraquinone content under these conditions is estimated to reach 19.8 mg/g. Upon re-fermentation under these optimized conditions, the measured anthraquinone content is 20.4 mg/g, which shows minimal deviation from the predicted value, affirming the validity and reliability of the model.

### 3.3 *In vitro* antioxidant cell experiments

#### 3.3.1 Safe concentrations of fermentation solution and H_2_O_2_ for cells

**Figure 3:**
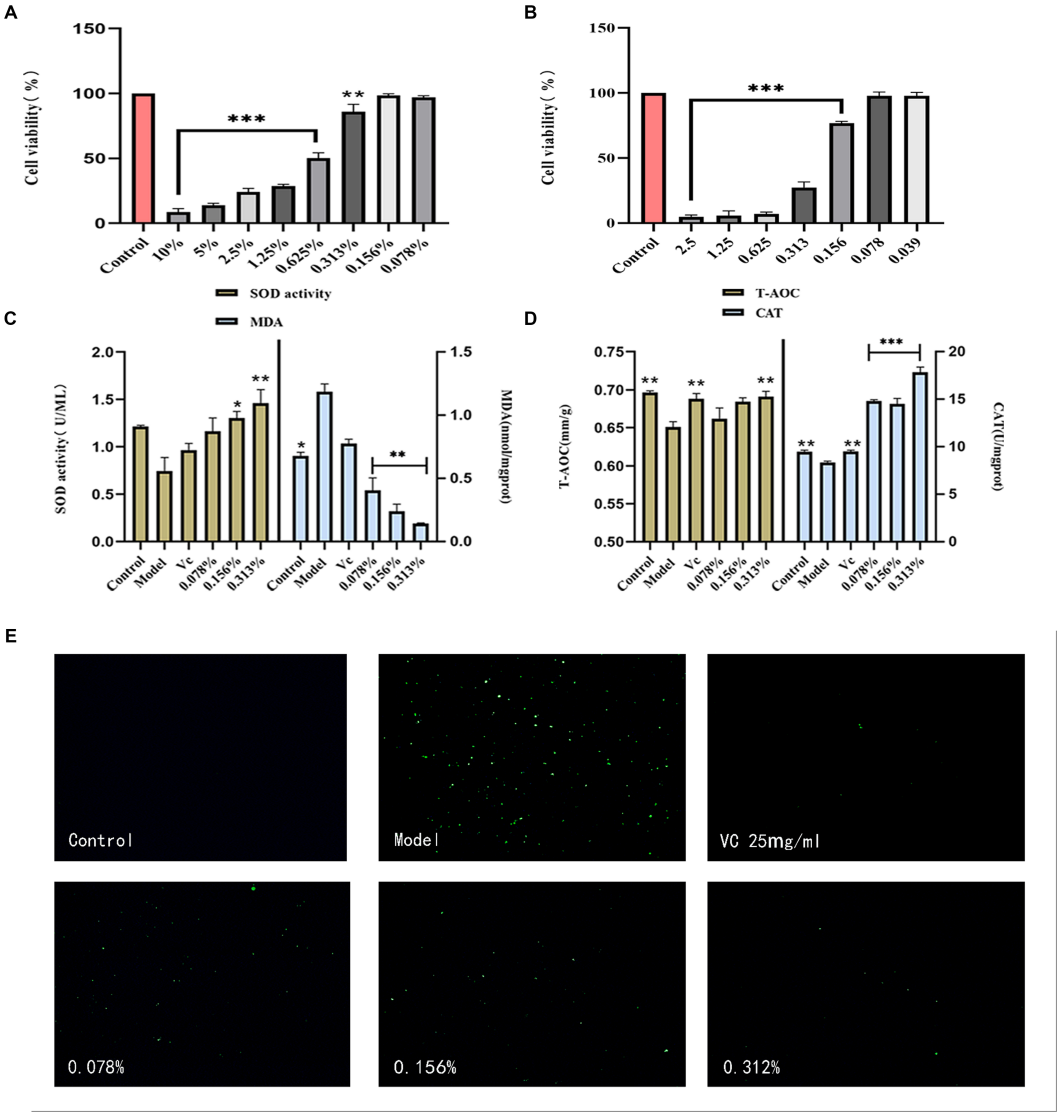
Results of antioxidant cell experiments. **(A)** HaCaT safety concentration, **(B)** H_2_O_2_ concentration, **(C)** Cellular SOD and MDA content, **(D)** Cellular T-AOC and CAT content, **(E)** Cellular ROS content.

The experiment assessed the cytotoxicity of the fermentation solution through the MTT assay, comparing it with the cell viability of the control group set at 100%. For fermentation solution concentrations below 0.313%, the cell survival rate exceeded 80% (*p* > 0.05), indicating minimal impact within this concentration range (Figure 3A). Therefore, fermentation solution concentrations of 0.078, 0.156, and 0.313% from cassia seed were designated as the low, medium, and high dosage groups.

H_2_O_2_ induces cell damage by promoting cell differentiation and affecting proteins (Zenin et al., 2022). In cellular models, H_2_O_2_ is commonly employed to induce oxidative damage or stress (Ransy et al., 2020). As shown in Figure 3B, as H_2_O_2_ concentrations increased, the cell survival rate began declining. When the concentration remained below 0.1mmol/L, the cell survival rate stayed above 75%. Therefore, 0.156 mmol/L of H_2_O_2_ was selected for establishing the oxidative damage model.

#### 3.3.2 Determination of cell lysate-related content

Superoxide dismutase (SOD) is considered a crucial antioxidant metal enzyme, and its activity reflects the extent of antioxidant capacity (Liang et al., 2023). As illustrated in Figure 3C, the SOD activity in the experimental model group was lower than that in the control group, indicating the successful establishment of a cellular oxidative model. Compared to the model group, the SOD activity in the fermentation solution group significantly increased. The lipid peroxidation marker, malondialdehyde (MDA) content, decreased with an increase in fermentation solution concentration (*p* < 0.01), and exhibited a better reduction in MDA compared to the positive VC group.

The T-AOC content was calculated by substituting the experimental data into the standard curve. The antioxidant effect of the fermentation solution was proportional to its concentration, and the T-AOC content in the 0.313% concentration group was comparable to the VC group (*p* < 0.01) (Figure 3D). Catalase (CAT) is a vital cellular enzyme that decomposes H_2_O_2_ and regulates reactive oxygen species (ROS) in cells (Okumoto et al., 2020). The fermentation solution significantly increased CAT content (*p* < 0.001) and surpassed the positive VC group, indicating that cassia seed fermentation solution possesses certain antioxidant efficacy.

#### 3.3.3 Cellular ROS content

Reactive oxygen species (ROS), as byproducts of aerobic metabolism, can lead to lipid peroxidation, DNA damage, and protein denaturation with their excessive accumulation (Lee and Song, 2021). In cellular experiments, hydrogen peroxide (H_2_O_2_), as a substance simulating oxidative stress, significantly increases intracellular ROS levels. In comparison to the model group, the impact of different concentrations of fermented cassia seed solution on ROS levels exhibits a dose-dependent relationship. As the concentration increases (Figure 3E), the inhibitory effect on ROS becomes more pronounced. Lowering ROS levels contributes to alleviating the adverse effects of oxidative stress on cells, indicating the potential antioxidant properties of fermented cassia seed solution at the cellular level.

### 3.4 *In vivo* antioxidant zebrafish experiments

#### 3.4.1 Safe concentrations of fermentation solution for zebrafish embryos

**Figure 4:**
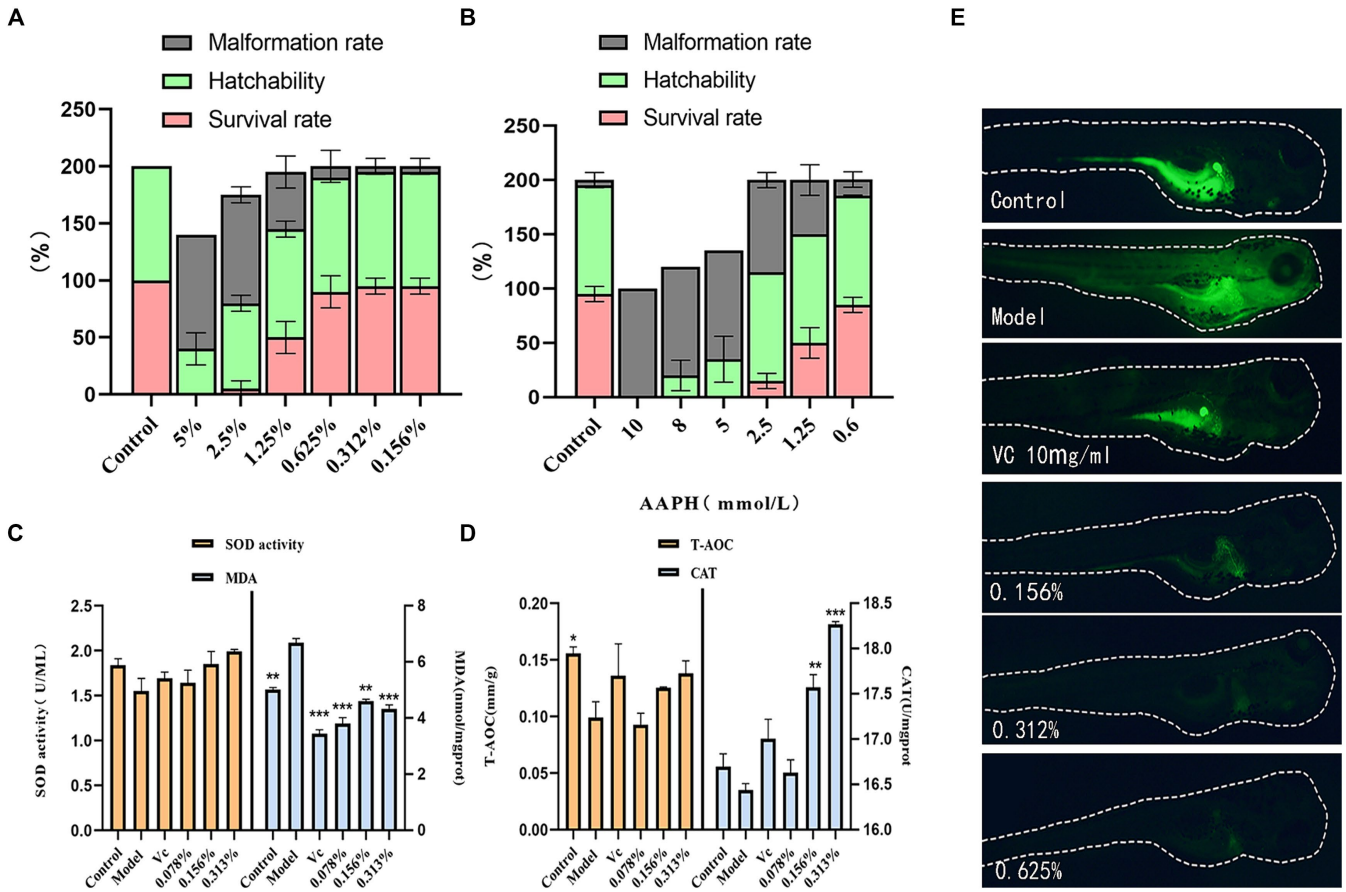
Results of antioxidant zebrafish experiments. **(A)** Effect of fermentation solution on zebrafish, **(B)** AAPH stimulation concentration, **(C)** Zebrafish SOD and MDA content, **(D)** Zebrafish T-AOC and CAT content, **(E)** Zebrafish ROS content.

Zebrafish, alongside other vertebrates, including humans, share a genetic homology of 70 ~ 80%. They are extensively utilized as animal models to evaluate drug toxicity due to their affordability, brief reproductive cycle, and visual characteristics (Li et al., 2022). The impact of cassia seed fermentation solution on embryos is evident in Figure 4A, within the concentration range of 0.156 to 0.625%, embryos demonstrate higher hatching and survival rates, coupled with a reduced incidence of abnormalities. AAPH, a potent water-soluble azo compound, is commonly employed as a free radical generator due to its ability to decompose, yielding nitrogen molecules and two carbon radicals (Zhu et al., 2023), It induces oxidative damage by attacking substances such as proteins. Embryo survival rates increase as the AAPH concentration decreases, particularly when below 2.5 mmol/L. At 0.6 mmol/L, both survival and hatching rates are higher, accompanied by a noticeable decrease in abnormalities. Thus, this concentration was selected as the benchmark concentration for oxidative stimulation (Figure 4B).

#### 3.4.2 Relevant levels in zebrafish

As illustrated in Figures 4C,D, the SOD content within zebrafish exhibits an upward trend with increasing concentrations of the fermentation solution, indicating a notable enhancement in the solution’s antioxidant capacity. Across all fermentation solution groups, the MDA content is lower than that in the model group (*p* < 0.01). It exhibits strong antioxidant properties against AAPH irritation. In the low-dosage group, the T-AOC content appears lower than that in the model group, potentially suggesting an unclear effect on enhancing zebrafish T-AOC content at this concentration. However, the medium and high-dosage groups display higher T-AOC levels than the model group (*p* > 0.05). The antioxidant prowess of cassia seed fermentation solution can be attributed to its antioxidant constituents, such as polyphenols, known to inhibit free radical activity, thereby mitigating tissue damage and aging (Salamatullah, 2022). This notably elevates the CAT content within zebrafish (*p* < 0.01).

#### 3.4.3 ROS content in zebrafish

This experiment utilized the fluorescent probe DCFH-DA for reactive oxygen species (ROS) detection (Zhang et al., 2024), enabling the observation of fluorescence intensity to determine ROS levels within zebrafish. The addition of copper sulfate can elevate ROS levels by inhibiting T-SOD, CAT activity, and glutathione (GSH) concentration (Fang et al., 2021). The results show significantly lower fluorescence intensity in the fermentation solution group compared to model groups. This suggests that the incorporation of cassia seed fermentation solution reduces the ROS content within zebrafish, demonstrating a distinct antioxidative effect (Figure 4E).

### 3.5 Anti-inflammatory cell experiments

#### 3.5.1 Safe concentration of fermentation solution for RAW264.7 cells

**Figure 5:**
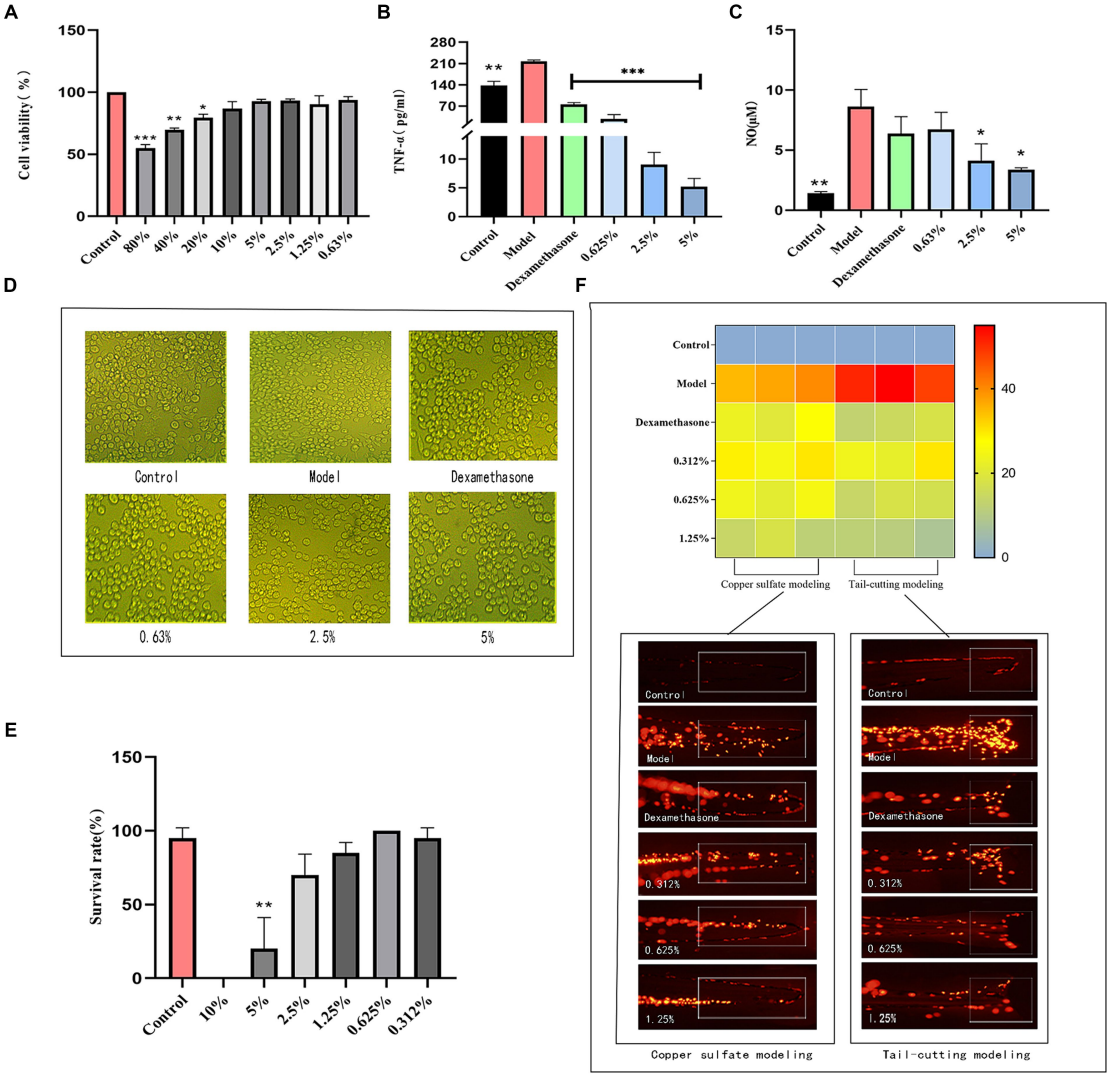
Results of anti-inflammatory experiments. **(A)** RAW264.7 safe concentration, **(B)** TNF-α content, **(C)** NO content, **(D)** Differences of RAW264.7 cells, **(E)** Survival of transgenic zebrafish, **(F)** Inflammation numbers and fluorescence figures.

When the fermentation solution concentration ranged from 0.63 to 10%, there was minimal impact on cell viability (*p* > 0.05). When the concentration was below 5%, the cell viability exceeded 90%. Therefore, the experimental investigation was conducted using concentrations ranging from 0.63 to 5% (Figure 5A).

#### 3.5.2 TNF-α and NO content

Inflammation represents an adaptive immune response by the host to damage or invading pathogens. Inflammatory cells within the immune system react to foreign substance intrusion and inflammatory stimuli, generating diverse inflammatory mediators (Zhao et al., 2023). In comparison to the control group, the model group stimulated with LPS exhibited elevated production levels of TNF-α and NO (Figures 5B,C). Nevertheless, fermented cassia seed solution effectively suppressed the generation of inflammatory factors, demonstrating a notable anti-inflammatory effect in a concentration-dependent manner. Stimulation of RAW264.7 macrophages by LPS triggers intracellular signal transduction, culminating in the release of inflammatory factors (Yuan et al., 2022). The cell differentiation manifested irregular polygonal shapes and protruding tentacles, upon the addition of cassia seed fermentation solution, the cells began to restore their normal morphology, transitioning to a more rounded and plump state, thereby exhibiting an improvement in cell differentiation (Figure 5D).

### 3.6 Anti-inflammatory zebrafish experiments

#### 3.6.1 Effect of fermentation solution on transgenic zebrafish

The influence of fermented cassia seed solution on transgenic zebrafish is depicted in Figure 5E. Survival rates remained relatively high within the concentration range of 0.312 to 2.5% (*p* > 0.05). However, the survival rate in the 2.5% concentration group dipped below 90%. Consequently, concentrations ranging from 0.312 to 1.25% of fermented solution were chosen for subsequent experiments.

#### 3.6.2 Copper sulfate and tail-cutting method induced inflammation model

Zebrafish macrophages and neutrophils constitute the early adaptive immune system (Peng et al., 2021). As illustrated in Figure 5F, zebrafish in the model group induced by copper sulfate exhibit a higher presence of inflammatory factors near the neural thalamus. Upon exposure to fermented cassia seed solution, a reduction in inflammatory factors was observed. At a fermentation solution concentration of 1.25%, the anti-inflammatory effect surpassed that of the positive group, significantly reducing the number of inflammatory cells. Inducing acute inflammation through tail amputation, inflammatory cells in the injured tail area were observed. In contrast to the model group, the fermented cassia seed solution group displayed a reduction of over twofold in the number of inflammatory cells (*p* < 0.01). Both methods effectively validate the significant anti-inflammatory effects demonstrated by fermented cassia seed solution, the effect is associated with its components, such as polyphenols and flavonoids (Rana et al., 2022), known for their inflammation-inhibiting properties.

### 3.7 Antibrowning cell experiment

#### 3.7.1 Safe concentrations of fermentation solution for B16-F10 cells

**Figure 6:**
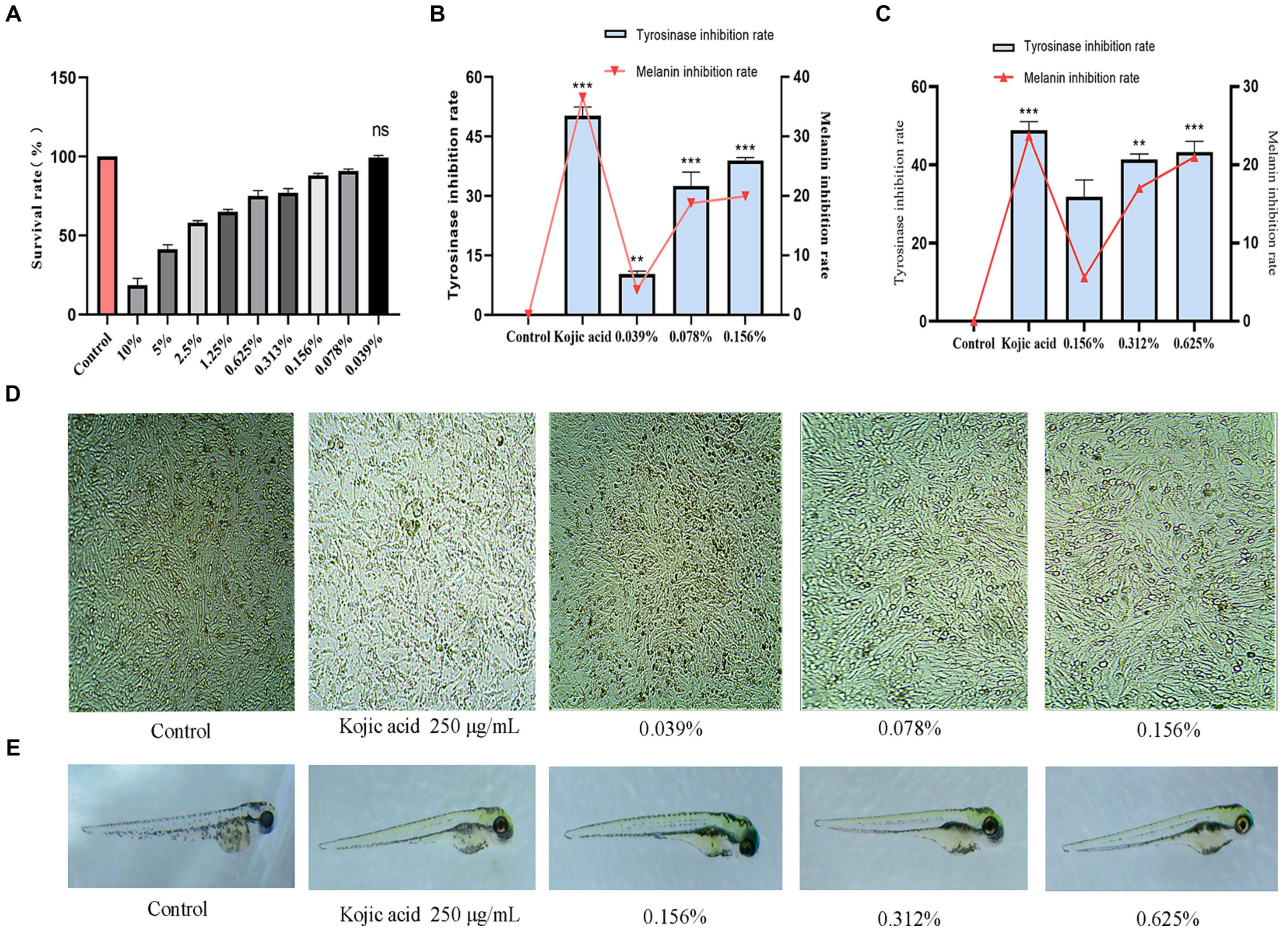
Results of antibrowning experiments. **(A)** Safe concentration of B16-F10 cells, **(B)** Inhibition rate of intracellular tyrosinase and melanin, **(C)** Inhibition rate of zebrafish tyrosinase and fish melanin, **(D)** Distribution of melanin in B16-F10 cells, **(E)** Distribution of melanin in zebrafish.

As the fermentation solution concentration decreases, the cell survival rate begins to increase. When the fermentation solution is diluted to 0.039%, no toxicity to cell growth is observed (*p* > 0.05). Therefore, an experimental range of concentrations from 0.039 to 0.156% was selected, during which the cell survival rate remained above 90% (Figure 6A).

#### 3.7.2 Intracellular tyrosinase and melanin inhibition

Tyrosinase serves as a pivotal enzyme in the catalysis of melanin production (Wang et al., 2024). In this experiment, the inhibitory effects of fermented cassia seed solution on tyrosinase and melanin were evaluated to assess its efficacy. Comparative to the control group, cassia seed fermentation solution exhibited a concentration-dependent inhibition of tyrosinase. At a concentration of 0.156%, the inhibition rate reached 40% (*p* < 0.001). Melanin inhibition at this concentration is 19.5% (Figure 6B). The cells were examined under a microscope after exposure to the fermentation solution, revealing a noticeable decrease in intracellular melanin synthesis as the concentration of the administered drug increased (Figure 6D). Enzymatic browning poses a significant challenge in the food industry (He et al., 2023), and cassia seed fermentation solution achieves antibrowning effects by inhibiting enzyme activity. If applied in the food sector, it could offer substantial benefits in terms of extending storage time, preventing appearance changes, preserving sensory characteristics, and maintaining nutritional value, thereby contributing to green and sustainable development.

### 3.8 Tyrosinase and melanin inhibition in zebrafish

A tyrosinase inhibitor competitively binds copper ions with tyrosinase, leading to tyrosinase inactivation and reducing the production of melanin (Tayier et al., 2021). At a fermentation solution concentration of 0.156%, the tyrosinase inhibition rate was 31.8% (*p* > 0.05). As the administered dosage increased to 0.625%, the inhibition rate escalated to 43.2% (*p* < 0.001). At this point, the melanin inhibition rate reached the optimal effect at 20.3% (*p* < 0.01) (Figure 6C). Compared to the control group, zebrafish in the fermentation solution group exhibited a significant reduction in melanin as observed in the lateral view (Figure 6E). The robust inhibitory effect of cassia seed fermentation solution on tyrosinase has been substantiated through cellular and zebrafish experiments, highlighting its potential as an enzyme inhibitor in preservatives, decolorants, and within the cosmetic and pharmaceutical industries.

### 3.9 Antibacterial experiment of cassia seed fermentation solution

#### 3.9.1 Minimum inhibitory concentration

**Figure 7:**
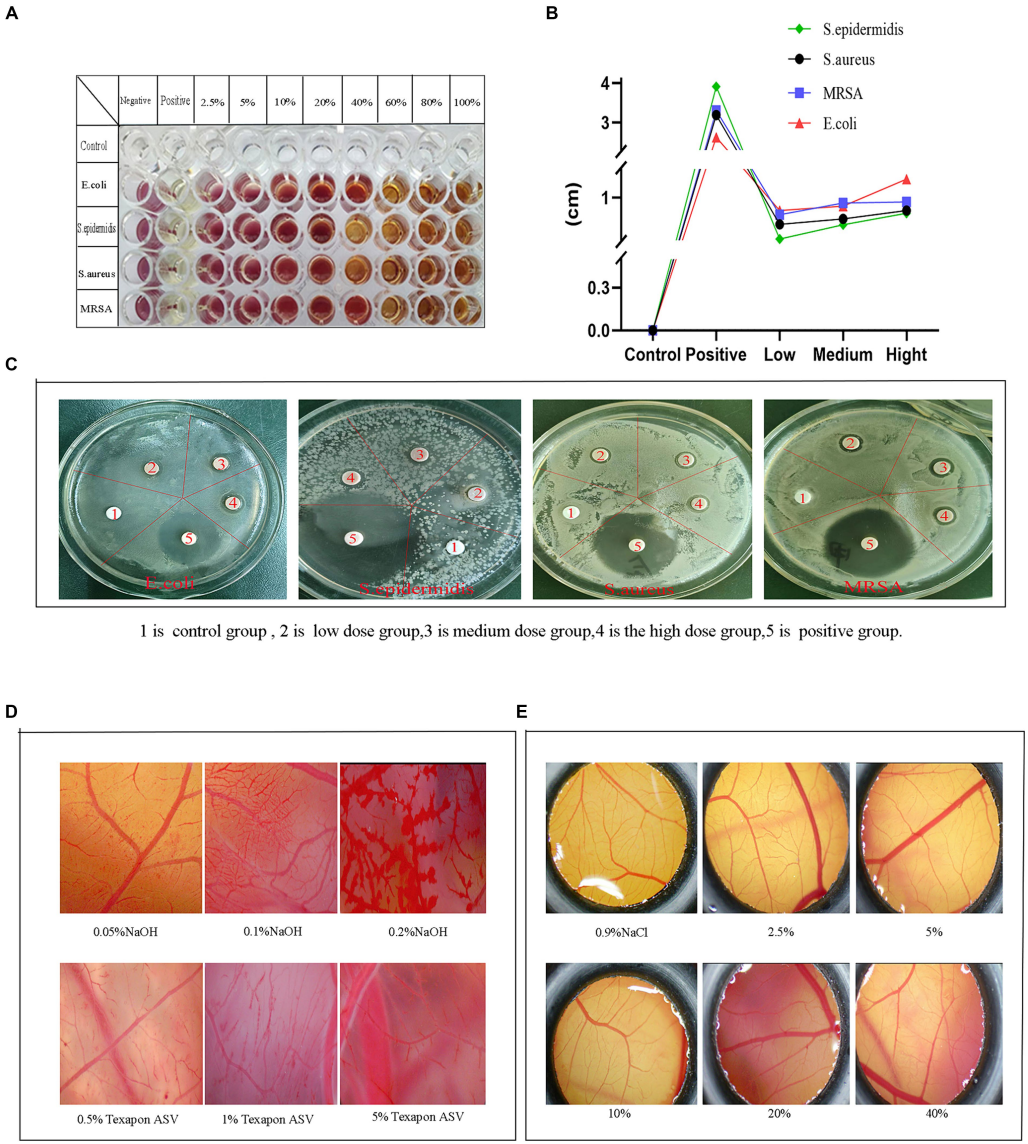
Bacteriostatic and safety test results. **(A)** Minimum inhibitory concentration, **(B)** Inhibitory diameter curve, **(C)** Circle of inhibition, **(D)** Effect of positives and reference on CAM vasculature, **(E)** Effect of the fermentation solution on the CAM.

TTC is a redox indicator, and dehydrogenases in living cells can reduce TTC to red triphenylmethyl filth (TPF). Leveraging this property, the minimum inhibitory concentration of the fermentation solution was established. Results indicated (Figure 7A). The minimum inhibitory concentration of the fermentation solution for *E. coli* was 60%. This concentration was categorized as the low concentration group, followed by 80% as the medium concentration group and 100% as the high concentration group. Similarly, for *Staphylococcus epidermidis*, concentrations of 40, 80, and 100% were categorized as low, medium, and high concentration groups, respectively. Likewise, for *Staphylococcus aureus*, concentrations of 40, 80, and 100% were grouped as low, medium, and high concentrations. Methicillin-resistant *Staphylococcus aureus* was categorized into low, medium, and high concentration groups at 60, 80, and 100%, respectively.

#### 3.9.2 Determination of the circle of inhibition

The excessive use of chemical preservatives in the food industry has led to a growing number of issues, including allergic reactions, toxicity, and antibiotic resistance, highlighting the urgent need to explore a safer alternative. In this experiment, the antimicrobial properties were assessed by examining the inhibitory effects of cassia seed fermentation solution on various bacterial strains (Figures 7B,C). No inhibitory zone was observed on the drug-sensitive paper discs in the control group. In contrast, the fermentation solution group demonstrated varying degrees of antibacterial activity, particularly exhibiting a relatively good inhibition effect against *Escherichia coli*, with a maximum inhibition zone diameter of up to 1.13 ± 0.12 cm. Cassia seed fermentation solution exhibits antibacterial effects by impacting bacterial outer membranes or protein synthesis mechanisms (Habiba et al., 2021), owing to its active components such as anthraquinones, flavonoids, and phenolic compounds.

### 3.10 Safety evaluation of fermentation solution

#### 3.10.1 Effects of positive control and reference control on CAM

The chorioallantoic membrane (CAM) of the chicken embryo is an extraembryonic membrane formed by the fusion of the allantois and the vascularized chorion (Ribatti, 2023). It serves as an *in vitro* method for evaluating eye irritation due to its cost-effectiveness and operational simplicity. This experiment aims to assess safety by examining the extent of stimulation induced by fermented cassia seed solution on the chicken embryo’s chorioallantoic membrane. The effect of a mixture of NaOH solution and fatty alcohol ether sodium sulfate (Texapon ASV) on CAM is illustrated in Figure 7D. The 0.5% Texapon ASV group resulted in slight bleeding and vascular dissolution, while the 1% Texapon ASV group showed moderate bleeding and vascular dissolution. The 5% Texapon ASV group displayed severe bleeding and vascular dissolution. The 0.05% NaOH group exhibited mild coagulation, the 0.1% NaOH group showed moderate coagulation, and the 0.2% NaOH group demonstrated severe coagulation.

#### 3.10.2 Effect of fermentation solution on CAM

Refer to the chorioallantoic membrane, which does not require administrative procedures approved by an animal experimentation ethics committee. When assessing the irritant potential of chemicals, CAM has been employed as an alternative to rabbit experiments (Yahya et al., 2023). The experiment involved applying various concentrations of the fermentation solution on the CAM and comparing it with the 0.9% NaCl group. The CAM treated with a 2.5 to 5% concentration of the fermentation solution displayed distinct and intact vascular structures and contours without any signs of bleeding, coagulation, or vascular dissolution. In contrast, the 10 to 40% fermentation solution groups exhibited minor capillary bleeding, vascular dissolution, and coagulation, while the vascular structure and contours within the CAM remained clear (Figure 7E). The ES score results can be found in Supplementary Table S4. Within an appropriate concentration range, cassia seed fermentation solution demonstrated minimal to no ocular irritation, suggesting its potential as a safe food or medical ingredient with promising prospects for further development.

Fermenting cassia seeds with microorganisms to increase their levels of active ingredients, thereby enhancing their pharmacological activity and efficacy, has bestowed upon cassia seeds greater medicinal value, particularly in terms of heightened antioxidant, anti-inflammatory, and other properties. This opens up new avenues and possibilities for processing and utilizing traditional Chinese medicinal materials, advancing their modern applications. Furthermore, producing medicinal or health foods with specific functions using fermented cassia seed solution can meet the growing demand for health and nutrition among people. This research serves as an example of innovative application of microbial fermentation technology in the processing of traditional Chinese medicinal materials, exploring and developing more fermentation methods for drugs to enhance their medicinal value and market competitiveness. In conclusion, research on fermented cassia seed solution is not only significant for the modern utilization of medicinal materials but also provides valuable insights and inspiration for the innovative application of microbial fermentation technology in the food and pharmaceutical industries.

## 4 Conclusion

The experimental results on HaCaT cells and zebrafish bodies indicate that cassia seed fermentation solution has a positive impact on oxidative stress. It inhibits the production of inflammatory factors in RAW264.7 cells and exhibits significant anti-inflammatory effects in a transgenic zebrafish inflammation model. Furthermore, the fermentation solution inhibits tyrosinase in B16-F10 cells and zebrafish bodies, thereby reducing melanin production. Its antibacterial properties allow it to suppress bacterial proliferation. The safety of this fermentation solution has been confirmed through chicken embryo experiments. Therefore, cassia seed fermentation solution holds paramount importance as a functional ingredient in research and development due to its positive effects on human health and environmental sustainability.

## Data availability statement

The original contributions presented in the study are included in the article/Supplementary material, further inquiries can be directed to the corresponding author.

## Ethics statement

The animal study was approved by Ethics Committee of Guangdong Pharmaceutical University. The study was conducted in accordance with the local legislation and institutional requirements.

## Author contributions

HX: Conceptualization, Formal analysis, Methodology, Resources, Validation, Visualization, Writing – review & editing. QG: Formal analysis, Software, Supervision, Writing – review & editing. WC: Conceptualization, Resources, Validation, Writing – review & editing. XM: Conceptualization, Resources, Validation, Writing – review & editing. ZG: Conceptualization, Formal analysis, Resources, Software, Visualization, Writing – review & editing. YZ: Conceptualization, Resources, Validation, Writing – review & editing. HL: Conceptualization, Project administration, Supervision, Validation, Writing – review & editing.
