# Supplementary material for: Mitigation of oxidative stress and inflammatory factors, along with the antibrowning and antimicrobial effects of cassia seed microbial fermentation solution
Source: Front Microbiol. 2024 May 9;15:1400505. doi: 10.3389/fmicb.2024.1400505 (PMC11112119; doi:10.3389/fmicb.2024.1400505)
Supplement: Supplementary file 1 [file Table_1.DOCX]

Supplementary Material

**Supplementary Table 1**. CAM experimental results judgement

| **Score** | **Condition** |
| --- | --- |
| No bleeding (0points) | No bleeding seen |
| Mild bleeding (1point) | Only slight bleeding observed in small blood vessels and minor blood loss |
| Moderate bleeding (2 points) | Bleeding observed in small and large blood vessels, accompanied by noticeable blood flow |
| Severe bleeding (3 points points) | Universal bleeding observed in blood vessels, accompanied by a significant amount of blood loss |
| No coagulation (0 points) | No signs of coagulation observed |
| Mild coagulation (1 point) | Slight coagulation inside and/or outside blood vessels and/or slight turbidity in the CAM membrane |
| Moderate coagulation (2 points) | Coagulation inside and/or outside blood vessels and/or moderate turbidity in the CAM membrane |
| Severe coagulation (3 points) | Coagulation inside and/or outside blood vessels and/or high turbidity in the CAM membrane |
| No vascular dissolution (0 points) | No signs of vascular dissolution observed |
| Mild vascular dissolution (1 point) | Only minor vascular dissolution observed |
| Moderate vascular dissolution (2 points) | Vascular dissolution observed in small and large blood vessels |
| Severe vascular dissolution (3 points) | Vascular dissolution observed in large and all blood vessels |

**Supplementary Table 2.** Plackett-Burman analysis of variance

| **Source** | **Sum of Squares** | **DF** | **Mean Square** | **F-Value** | **p-value** | **Saliency List** |
| --- | --- | --- | --- | --- | --- | --- |
| Model | 55.72 | 5 | 11.14 | 23.92 | 0.0007 | significant |
| A- Lactose concentration | 0.3536 | 1 | 0.3536 | 0.7591 | 0.4171 | 4 |
| B- Inoculation amount | 7.33 | 1 | 7.33 | 15.74 | 0.0074 | 2 |
| C- Fermentation temperature | 44.16 | 1 | 44.16 | 94.80 | <0.0001 | 1 |
| D- Material-liquid ratio | 0.1323 | 1 | 0.1323 | 0.2840 | 0.6132 | 5 |
| E- Fermentation time | 3.74 | 1 | 3.74 | 8.03 | 0.0298 | 3 |
| Residual | 2.80 | 6 | 0.4658 |  |  |  |
| Cor Total | 58.51 | 11 |  |  |  |  |

**Supplementary Table 3.** Box-Behnken design and results

| **Source** | **Sum of Squares** | **DF** | **Mean Square** | **F-Value** | **p-value** | **Significance** |
| --- | --- | --- | --- | --- | --- | --- |
| Model | 69.74 | 9 | 7.75 | 64.51 | <0.0001 |  |
| A-Fermentation Time | 0.9870 | 1 | 0.9870 | 8.22 | 0.0241 |  |
| B- Inoculation amount | 3.82 | 1 | 3.82 | 31.82 | 0.0008 |  |
| C- Fermentation time | 10.58 | 1 | 10.58 | 88.08 | <0.0001 |  |
| AB | 0.2970 | 1 | 0.2970 | 2.47 | 0.1598 |  |
| AC | 1.44 | 1 | 1.44 | 11.99 | 0.0105 |  |
| BC | 3.13 | 1 | 3.13 | 26.08 | 0.0014 |  |
| A^2^ | 24.40 | 1 | 24.40 | 203.13 | <0.0001 |  |
| B^2^ | 5.07 | 1 | 5.07 | 42.20 | 0.0003 |  |
| C^2^ | 15.36 | 1 | 15.36 | 127.85 | <0.0001 |  |
| Residual | 0.8408 | 7 | 0.1201 |  |  |  |
| Lack of Fit | 0.6569 | 3 | 0.2190 | 4.76 | 0.0828 | Not Significance |
| Pure Error | 0.1839 | 4 | 0.0460 |  |  |  |
| Cor Total | 70.58 | 16 |  |  |  |  |
